# The association between spirometry measurement quality, cognitive function, and mortality

**DOI:** 10.1186/s13690-025-01660-x

**Published:** 2025-07-01

**Authors:** Consuelo Quispe-Haro, Tatyana Court, Magdalena Kozela, Abdonas Tamosiunas, Nadezda Capkova, Hynek Pikhart, Martin Bobák

**Affiliations:** 1 RECETOX, Faculty of Science, Masaryk University, Kotlarska 2, Brno, Czech Republic; 2 Department of Epidemiology and Population Studies, Institute of Public Health, Faculty of Health Sciences, Jagiellonian University Medical College, Kraków, Poland; 3 Institute of Cardiology, Lithuanian University of Health Sciences, Kaunas, Lithuania; 4 National Institute of Public Health, Prague, Czech Republic; 5 Research Department of Epidemiology and Public Health, University College London, London, UK

**Keywords:** Aging, Cognition, Lung function, Mortality, Quality of spirometry

## Abstract

**Background:**

Population studies that assess lung function usually exclude results of individuals with poor-quality measurements, which often means excluding many subjects. Impaired cognition is frequently associated with poor-quality spirometry; excluding such subjects may introduce a selection bias in studies with lung function as either outcome or exposure. We investigated the association between poor-quality spirometry and impaired cognitive function and whether poor-quality spirometry is associated with future mortality risk independently of cognitive function.

**Methods:**

We used data from a prospective cohort in three Central and Eastern European countries; 12,087 individuals aged 45–75 years (54% females) with complete information on variables of interest were included. Standard memory, verbal fluency, and executive cognitive domain tests were converted into latent variable z-scores and divided into quartiles. Spirometry tests were classified into two categories based on repeatability criteria: good- (71%) vs. poor-quality spirometry (29% of participants). Those with good-quality spirometry were further classified, using forced vital capacity (FVC) and forced expiratory volume in the first second (FEV_1_), as healthy spirometry (63%) or impaired spirometry (8%). Multinomial logistic regression was used to assess the association between poor-quality spirometry and cognitive function, and a Cox proportional regression was used to analyze the risk of total mortality over a 17-year follow-up.

**Results:**

After controlling for a range of covariates, higher cognitive function predicted lower odds of poor-quality spirometry. In the highest cognitive function quartile, compared with the lowest quartile, the odds ratio of poor-quality spirometry was 0.82 (95%CI: 0.72–0.92). Impaired spirometry was associated with higher mortality risk even after adjusting for cognition (adjusted hazard ratio 1.63, 95%CI: 1.45–1.84), but mortality risk was similar in subjects with poor- vs. good-quality (HR 1.02, 95%CI: 0.93–1.10).

**Conclusion:**

Higher cognitive function was associated with a lower risk of poor-quality spirometry. The lack of independent association of poor-quality spirometry with mortality suggests that excluding poor-quality spirometry measurements from analyses is unlikely to introduce a major bias. However, discarding poor-quality spirometry from epidemiological analyses might imply the exclusion of vulnerable subjects. These findings should be confirmed in future studies representing other populations.

**Supplementary Information:**

The online version contains supplementary material available at 10.1186/s13690-025-01660-x.

**Table:** 

Text box 1. Contributions to the literature	
• There is good evidence that lung function and cognition are positively associated, and these markers are prospectively associated with mortality • Subjects with lower cognitive functions are more likely to provide poor-quality spirometry data • These results suggest that excluding poor-quality spirometry data is unlikely to introduce major bias in estimating mortality risk	

## Background

Spirometry is a well-established (and often overlooked) biomarker of health and future risk [1, 2]. However, the measurement is not always performed correctly. Poor-quality spirometry is defined as the inability of people to perform a respiratory test that fulfills the acceptability and repeatability criteria [3]. During a spirometry examination, individuals first receive a series of instructions on how to perform the test. Three correctly performed readings are needed to evaluate the pulmonary capacity; however, individuals must repeat the test three to eight times to meet acceptability and repeatability criteria [3]. Around 10% of people are usually not able to reach these standards [4], and the information on their pulmonary capacity remains unreliable. This may potentially introduce a selection bias if spirometry measurements that failed to satisfy acceptability and repeatability criteria are excluded from statistical analyses.

A spirometry test requires a comfortable environment, a well-trained technician, and the ability of the person to understand and execute a list of instructions. This last point is of particular interest to this paper. Previous studies report that older people with compromised cognition may be unable to perform good-quality spirometry [5–9]. However, another study found that, after controlling for covariates, an impaired cognitive function measured using the Mini-Mental State Exam (MMSE) was not an independent predictor of poor-quality spirometry [10]. Additionally, as people age, the number of comorbidities is likely to increase, which can also lead to poor spirometry performance.

Several other risk factors might explain the association between cognitive function and poor-quality spirometry. Bellia et al. [9] reported that among individuals over 65, motor and sensory deficits, depression, dementia, and malnutrition were associated with poor-quality spirometry. Other studies have highlighted demographic and socioeconomic influences. For instance, Tan et al. [11] found that female sex and younger age were associated with higher odds of producing good-quality spirometry, while education had no significant effect. In contrast, in the United States people with lower income, male sex, and African American ethnicity were associated with poor-quality spirometry measurement [12]. Although these findings offer insight into potential contributing factors, results across studies have been inconsistent. Possible reasons for this include heterogeneity in study populations, variations in spirometry protocols and quality criteria, differences in how cognitive function was assessed, and the presence of unmeasured or residual confounding.

In addition to the association between cognition and poor-quality spirometry, there is evidence that cognitive function is also associated with impaired lung function [13, 14]. As most studies of poor-quality spirometry compared poor-quality with good-quality spirometry, which includes both healthy and impaired lung function, the comparison may not be perfect.

Nevertheless, there is no evidence whether poor-quality spirometry is associated with future health outcomes, while there is extensive evidence that low cognitive function [15, 16] and impaired lung function [17] are associated with a higher risk of mortality.

In this study, we investigated the associations between cognitive function and poor-quality spirometry, and whether poor-quality spirometry is associated with future mortality both before and after controlling for cognitive function.

## Methods

### Study design

The HAPIEE study (Health, Alcohol, and Psychosocial Factors in Eastern Europe) [18] is a population-based epidemiological study carried out in urban centers of Poland, Lithuania, and the Czech Republic. The target population included adults aged 44 to 75 years at baseline between 2002 and 2008.

### Ethical approval

The study was approved by the Ethics Committees at University College London, UK, and in each participating center. All participants provided written informed consent prior to data collection.

### Inclusion and exclusion criteria

Only participants who underwent cognitive and lung function assessments, and for whom complete information was available on key covariates, were included in the present analysis. Individuals who were not invited for cognitive testing, refused examination in the clinic and/or spirometry testing, or lacked consent for mortality linkage were excluded.

### Tools development and training

Standardized questionnaires and examination protocols were developed jointly across study sites. Study personnel were trained centrally to ensure consistency in data collection procedures across countries; a relatively large number of nurses (more than 10) were involved in data collection, including spirometry testing.

### Quality assurance measures

Health personnel were centrally trained to collect health questionnaires, anthropometric information, cognitive function tests, and spirometry tests. Quality assurance included regular supervision of fieldwork, calibration of equipment, and periodic data audits.

### Spirometry

**Fig. 1:**
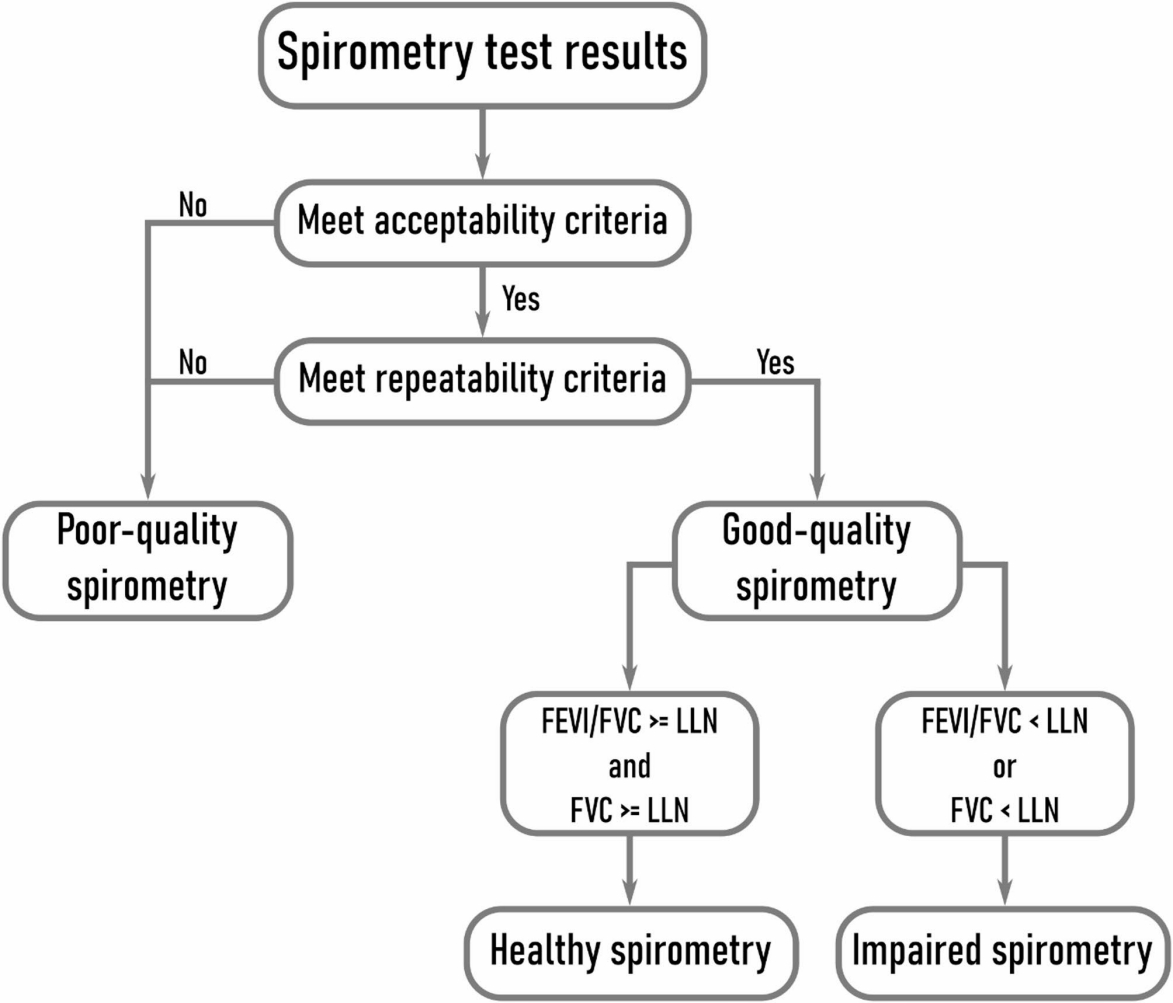
Flowchart illustrating the classification process for spirometry results into specific categories

The lung function was assessed using a Micro-Medical Microplus spirometer at the examination center by trained personnel. Only pre-bronchodilator measurements were obtained, as post-bronchodilator testing was not included in the study protocol. Spirometry quality evaluation followed the acceptability and repeatability criteria of the American Thoracic Society (ATS) and the European Respiratory Society (ERS) [3]. To meet acceptability criteria, examiners visually inspected the spirograms to achieve an expiration longer than 6 s and a good peak expiratory flow with no cough during the first second of the test. Three to eight acceptable tests were required. If the participant was unable to perform at least three acceptable tests, it was classified as poor-quality spirometry. Repeatability refers to the difference between the two highest values of the forced vital capacity (FVC) and the two highest values of forced expiratory volume in the first second (FEV_1_). The difference must be lower than 150 ml to fulfill the repeatability criteria [3]. Spirometry results were classified as poor-quality if participants failed the repeatability criteria in either FVC or FEV_1_, otherwise, the test was considered good-quality (Fig. 1).

Individuals with good-quality spirometry were further categorized as healthy or impaired spirometry based on the algorithm proposed by Pellegrino et al. [19] and the latest recommendations for the interpretation of spirometry [20] (Fig. 1). Participants were categorized as healthy spirometry when they achieved a FEV_1_/FVC ratio and an FVC higher than the lower limits of normality (LLN), otherwise, it was considered impaired spirometry. The LLN for the FEV_1_/FVC ratio and FVC represent the lower fifth percentile of the reference values, which are specific for every individual based on their age, sex, and height. We used the Global Lung Function Initiative LLN formulas as recommended by the European Respiratory Society and American Thoracic Society [21].

### Cognitive function

Cognitive function was tested using three standard tasks that evaluated different aspects of cognition. *Immediate and delayed verbal memory* consisted of a list of 10 words listened to by the participant and immediately asked to remember as many words as possible in two minutes. The task was repeated three times and recorded in a range from zero to ten, depending on the number of correct words. After five minutes, the participants were asked again to recall as many words as possible. In the meantime, *semantic and verbal fluency* were evaluated by asking the participants to name as many animals as possible in one minute. Then, *speed and concentration* were tested using a sheet with random letters of the alphabet in which participants had to cross out letters P and W. These tests have been used to measure the onset and progression of cognitive decline and dementia in longitudinal population studies [22].

The scores of each cognitive test were standardized to the respective z-scores to give a mean of zero and a standard deviation of one. The six z-scores were used to predict a latent variable called “Cognition”, where higher scores represent better cognitive function. First, we checked the Spearman correlations of the six cognitive tests [see Additional file 1]. Second, we employed confirmatory factor analysis to calculate the latent variable “Cognition” and extracted the predicted factor [23]. Finally, we categorized the predicted factor into quartiles (4). Quartiles of cognition were derived from the full sample with available cognitive data. Due to missing data in other variables, the final analytic sample included an unequal distribution of participants across cognition quartiles.

### Mortality

Individuals were followed from the examination date until August 2017 in Poland, April 2019 in Lithuania, and January 2021 in the Czech Republic. Follow-up time therefore varied by country, with a maximum duration of 17 years. Dates of deaths were obtained from the national death registry. All-cause mortality was used in the analyses.

### Covariates

From the health questionnaires, we used a set of covariates that have been previously associated with worst pulmonary outcomes and mortality [24, 25]: age, sex, study area, smoking, education, diagnosed or hospitalized for stroke, and diagnosed or hospitalized for respiratory disease. Smoking was coded as a dummy variable between those who never smoked (0) and past and current smokers (1). The highest level of education accomplished was divided into three categories: no education plus primary education (0), secondary education (1), and tertiary education (2). If a person was previously diagnosed or hospitalized for stroke was coded as one (1), otherwise zero (0). Identical coding was used if diagnosed or hospitalized for respiratory disease. The BMI is a derived variable using the conventional formula weight(kg)/height(m)^2^.

### Statistical analysis

Analyses were conducted using Stata/IC 16.1. First, we performed multinomial logistic regression to investigate the associations between cognitive function and the categories of healthy, impaired, and poor-quality spirometry, with healthy spirometry as the reference group. In model 1, cognitive function was adjusted for age, sex and study area, and in model 2 cognitive function was additionally adjusted for a range of covariates. Results are presented as odds ratios (OR) and 95% confidence intervals (CI). Second, we used logistic regression to analyze associations of cognitive function with good- vs. poor-quality spirometry measurement.

Third, we calculated mortality rates (deaths per 1000 person-years) for each spirometry category. We used Cox proportional hazards regression models to assess the association between spirometry categories and all-cause mortality and estimate hazard ratios (HRs) at 95% CI. Three models were constructed: model 1 was adjusted for age and sex; Model 2 was adjusted for age, sex, and cognitive function as a continuous variable, and Model 3 was adjusted for all covariates including cognitive function as a continuous variable. Cox regression analyses were also performed for the association of mortality with good- vs. poor-quality spirometry measurement.

## Results

**Fig. 2:**
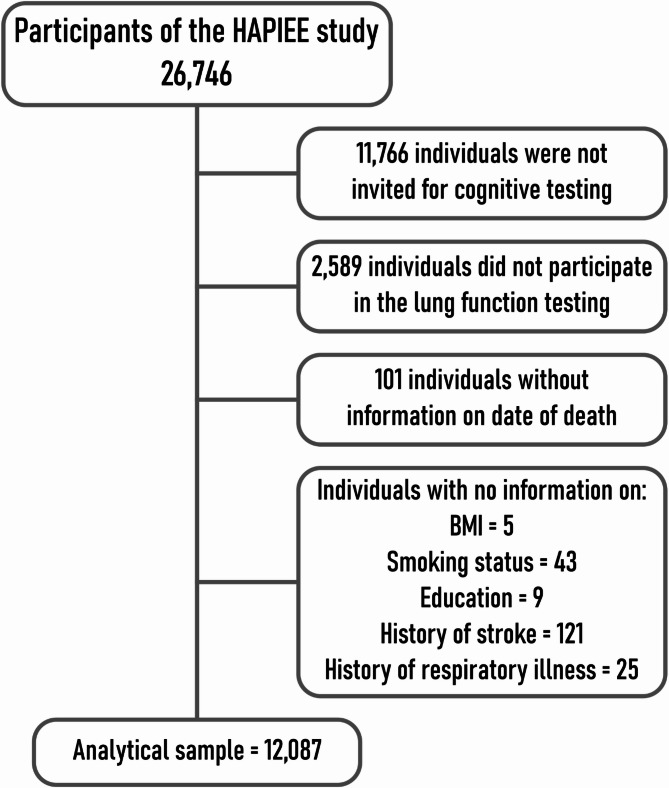
Participants of the HAPIEE study excluded from the analysis

A total of 26,746 individuals responded to a health questionnaire and underwent a medical examination. From these, 11,766 individuals were not invited to the cognitive function examination and therefore were excluded as ineligible from the final sample (Fig. 2) due to logistic and funding constraints that restricted the cognitive assessment to a subsample of the total cohort. Out of the remaining people, 2,589 declined to participate in the lung function testing. Additionally, 101 individuals did not provide consent to check the mortality status in the death registry. A smaller number of individuals were excluded due to missing data on key variables such as body mass index (BMI, *n* = 5), smoking status (*n* = 43), level of education (*n* = 9), and previous diagnosis/hospitalization due to stroke (*n* = 121) or respiratory disease (*n* = 25). These data were missing mostly because of item non-response in the questionnaire.

The analytical sample was restricted to individuals with complete (non-missing) information on spirometry, cognition, mortality, and covariates. It included 12,087 individuals, of whom 29% had poor-quality spirometry and 54% were females. The last day of follow-up was the 30th of January 2021. In total, 2,598 (21.49%) individuals died during this time. The characteristics of participants are presented in Table 1. Age, sex, BMI, smoking status, education, history of stroke, history of respiratory disease, mortality, and cognitive function were classified into three spirometry categories: healthy, impaired, and poor-quality spirometry.

The category of impaired spirometry had the highest prevalence of people that reported being past or current smokers (57%), primary education (20%), previous diagnosis or hospitalization for stroke (6%), previous diagnosis or hospitalization for respiratory diseases (27%), and mortality (37%). By contrast, most people with healthy spirometry are in the highest quartile of cognitive function (30%), while most people with impaired spirometry (29%) are in the lowest quartile of cognitive function.

**Table 1:** Characteristics of participants of the HAPIEE study between 2002–2008 by spirometry quality

	Good-Quality spirometry				Poor-Quality spirometry	
	Healthy spirometry *n* = 7 633 (63%)		Impaired spirometry *n* = 973 (8%)		*n* = 3 481 (29%)	
	N	% (or SD as indicated)	N	% (or SD as indicated)	N	% (or SD as indicated)
**Sex**						
Males	3 325	43.56	467	48.00	1 794	51.54
**Age**, years, mean, SD	61.26	7.01	62.20	6.35	61.84	6.91
**BMI**, kg/m^2^, mean, SD	28.83	4.85	30.70	5.61	29.23	5.16
**Smoking**						
Past or current smoker	3 267	42.80	553	56.83	1 519	43.64
**Education**						
Primary	886	11.61	198	20.35	487	13.99
Secondary or Vocational	3 605	47.23	506	52.00	1 640	47.11
Tertiary	342	41.16	269	27.65	1 354	38.90
**Hospitalized or diagnosed for stroke**						
Yes	244	3.20	59	6.06	160	4.60
**Hospitalized or diagnosed for Chronic respiratory disease**						
Yes	1 109	14.53	265	27.24	527	15.14
**Mortality**						
Yes	1 430	18.73	461	37.10	807	23.18
**Cognitive function**						
Cognition, mean, SD	0.11	0.64	-0.11	0.70	-0.002	0.67
Q 1 (lowest)	1 448	18.97	285	29.29	835	23.99
Q 2	1 742	22.82	266	27.34	915	26.29
Q 3	2 137	28.00	220	22.61	844	24.25
Q 4 (highest)	2 306	30.21	202	20.76	887	25.48
**Study area**						
Poland	1 008	13.52	300	26.11	577	16.58
Lithuania	4 426	59.36	467	40.64	1 972	56.65
Hradec	175	2.35	54	4.70	199	5.72
Jihlava	391	5.24	61	5.31	158	4.54
Karvina	389	5.22	93	8.09	207	5.95
Liberec	425	5.70	51	4.44	152	4.37
Usti nad Labem	642	8.61	123	10.70	216	6.21

HAPIEE: Health, Alcohol, and Psychosocial Factors in Eastern Europe study, conducted between 2002 and 2008 in urban centers of Poland, Lithuania, and the Czech Republic

Table 2 shows the OR from the multinomial logistic regression model, where the outcome variable is spirometry with three categories. In the age-sex adjusted model, higher cognitive function predicted lower odds of being in the impaired (OR: 0.47, 95%CI: 0.38–0.57) and poor-quality (0.74, 0.65–0.83) spirometry categories compared to healthy spirometry. Similarly, there was a strong association of cognitive function with poor- (vs. good-) quality spirometry measurement; the highest quartile of cognitive function was associated with lower odds of poor-quality spirometry (0.81, 0.71–0.91). Additional adjustments for other covariates (model 2) somewhat reduced these estimates but both remained strong and statistically significant. Aside from cognitive function, we found that being female was associated with a lower risk of poor-quality spirometry, while previous diagnosis or hospitalization for stroke increased the risk of poor-quality spirometry [see Additional file 2].

**Table 2:** Associations between cognitive function and spirometry quality in participants of the HAPIEE study between 2002–2008

	Cognitive function Model 1, OR (95% CI)				Cognitive function Model 2, OR (95% CI)			
	First quartile (*n* = 2 568)	Second quartile (*n* = 2 923)	Third quartile (*n* = 3 201)	Fourth quartile (*n* = 3 395)	First quartile (*n* = 2 568)	Second quartile (*n* = 2 923)	Third quartile (*n* = 3 201)	Fourth quartile (*n* = 3 395)
**Multinomial regression** (healthy spirometry is the reference group)								
Impaired vs. healthy spirometry	1.00	0.82 (0.68–0.97)	0.59 (0.48–0.70)	0.56 (0.45–0.68)	1.00	0.92 (0.76–1.09)	0.70 (0.57–0.85)	0.70 (0.56–0.86)
Poor-quality vs. healthy spirometry	1.00	0.93 (0.82–1.04)	0.70 (0.62–0.79)	0.72 (0.62–0.81)	1.00	0.95 (0.84–1.07)	0.73 (0.64–0.83)	0.75 (0.65–0.86)
**Logistic regression** (good-quality spirometry is the reference group)								
Poor- vs. good-quality spirometry	1.00	0.96 (0.85–1.08)	0.76 (0.67–0.86)	0.78 (0.68–0.88)	1.00	0.97 (0.86–1.09)	0.77 (0.68–0.87)	0.79 (0.69–0.90)

Model 1 adjusted for age, sex and study area

Model 2 adjusted for age, sex, study area, BMI, smoking status, education, hospitalized or diagnosed for respiratory disease, hospitalized or diagnosed for stroke

HAPIEE: Health, Alcohol, and Psychosocial Factors in Eastern Europe study, conducted between 2002 and 2008 in urban centers of Poland, Lithuania, and the Czech Republic

Table 3 shows the hazard ratios (HR) of mortality between categories of spirometry. In total, 2,598 individuals died during the follow-up. The mortality rate per 1000 person-years was the lowest among subjects with healthy spirometry, and highest among people with impaired spirometry. The Cox proportional hazard regression showed that, compared to the healthy spirometry group, poor-quality spirometry was associated with HR 1.15 when controlled for age, sex and area, which was attenuated to 1.12 when additionally adjusted for cognitive function, and to 1.11 in the fully adjusted model. When using the dichotomous variable of the quality of spirometry, poor-quality spirometry itself did not predict future mortality, either before (HR 1.03, CI 0.94–1.12) or after adjustment (HR 1.02, CI: 0.93–1.10).

**Table 3:** Hazard ratios for all-cause mortality by baseline spirometry category in participants of the HAPIEE study

	No. of persons	No. of deaths	Person years of follow-up	Deaths per 1000 person-years	Model 1 HR (95% CI)	Model 2 HR (95% CI)	Model 3 HR (95% CI)
**Three categories of spirometry**							
Healthy spirometry	7633	1 430	95 701	14.94 (14.18–15.73)	1.00	1.00	1.00
Impaired spirometry	973	361	11 258	31.97 (28.83–35.45)	2.04 (1.83–2.27)	1.94 (1.74–2.16)	1.67 (1.49–1.86)
Poor-quality spirometry	3 481	806	42 908	18.78 (17.53–20.12)	1.19 (1.09–1.30)	1.16 (1.06–1.26)	1.14 (1.04–1.24)
**Two categories of spirometry**							
Good-quality spirometry	8 606	1 791	106 959	16.73 (15.97–17.52)	1.00	1.00	1.00
Poor-quality spirometry	3 481	807	42 908	18.78 (17.53–20.12)	1.04 (0.95–1.13)	1.02 (0.93–1.10)	1.02 (0.94–1.11)

Model 1 adjusted for age, sex, and study area

Model 2 adjusted for age, sex, study area, and cognitive function

Model 3 adjusted for age, sex, study area, BMI, smoking status, education, hospitalized or diagnosed for respiratory disease, hospitalized or diagnosed for stroke, and cognitive function

HAPIEE: Health, Alcohol, and Psychosocial Factors in Eastern Europe study, conducted between 2002 and 2008 in urban centers of Poland, Lithuania, and the Czech Republic. Participants were followed for mortality until 2017–2021 depending on country

In a sensitivity analysis, we tested the robustness of cognitive function using the full sample size [see Additional file 3], which showed identical results to Table 2. Finally, the composite score of cognitive function showed stronger power than individual domains of cognition [see Additional file 4].

## Discussion

This study investigated the relationship between cognitive function and poor-quality spirometry tests, as well as the potential association of poor-quality spirometry tests with future mortality. The motivation for these analyses was two-fold. First, to confirm whether lower cognition is associated with a higher likelihood of poor-quality spirometry. Second, to see whether this association, if it exists, may introduce a selection bias in analyses of lung functions with mortality outcomes if subjects who provided poor-quality spirometry measurements are excluded from the analyses. We found that cognitive function was significantly inversely associated with the likelihood of having impaired or poor-quality spirometry. However, the longitudinal association of poor-quality spirometry with mortality was weak (or non-existent) and it was not materially attenuated by adjustment for cognitive function. At least in this cohort study, excluding “poor-quality” spirometry from analyses did not seem to lead to a major bias in analyses of all-cause mortality.

The results on associations of spirometry with both cognitive function and future mortality risks were as expected and consistent with the previous body of evidence [1, 13, 14, 16, 26, 27]. However, despite the simultaneous association between the quality of spirometry measurement and cognitive function and between cognitive function and mortality, there was no detectable association between poor-quality spirometry measurement and mortality. This observation suggests that the strength of the association of cognitive function with poor-quality measurement is too weak to translate into an association of poor-quality measurement with mortality. Results may look different for health outcomes other than mortality, but our data suggest that excluding poor-quality measurements from analyses is unlikely to produce a major selection bias that would change the effect estimates for spirometry.

Our findings align with some of the existing evidence. Previous studies have reported that older people with compromised cognitive function may struggle to perform good-quality spirometry [5–7]. However, some studies found no association associations between poor-quality spirometry and cognitive function [10]. Inconsistencies might arise from differences in the sample characteristics composition, measurements of cognitive function, and available information to evaluate the quality of spirometry. For instance, our study’s mean age was 62, while in other studies the mean age was 73 to 75 [6, 8, 9] and 84 [7, 10], which are more likely to have a higher prevalence of cognitive deficits and therefore fail the test; however, Haynes [28] found that individuals older than 80 years had similar quality of spirometry compared to individuals aged 40 to 50. Turkeshi et al. [10] included spirograms to classify the quality of spirometry into four categories, but spirograms were not available in our dataset for a secondary analysis. Finally, some authors used separate domains of cognitive function instead of a composite score. When specific cognitive domains were tested, such as the executive function assessed by the MMSE, the findings were negative [7]. Our results suggest that cognitive function remains a significant predictor when using a composite score derived from testing multiple domains [See Additional file 4].

In addition to differences in cognitive assessment methods, differences in statistical modeling approaches or differences in comparison groups and classification of healthy and impaired spirometry may play a role in the inconsistencies in previous studies. Our study sample was younger than many samples previously explored; while this confirms that lower cognitive function could predict poor-quality spirometry, differences in age groups between studies may also affect the consistency of results. Similar to other studies, we found that age was not a determinant of poor-quality spirometry [28]. Previous research has also identified various sociodemographic factors associated with spirometry performance [11, 12]. Our findings corroborate that females are less likely to have poor-quality spirometry, while education showed no detectable effect.

One issue to consider is the very high proportion (29%) of subjects classified as providing poor-quality spirometry measurement. This is much higher than the approximately 10% reported by many previous studies. For instance, Berresheim et al. [4] found a 10% poor-quality spirometry in two occupational settings with a median age of 41 and 20 years. On the other hand, Queiroz and colleagues [6] reported that 25% of adults older than 61 years performed a poor-quality spirometry, although the repeatability criteria threshold was set at 200mL instead of the 150mL currently recommended [6]. This is expected as more strict criteria should increase the number of people classified as poor-quality. In this sense, a group in Italy [9] that used a cut-off of 200mL found that, in healthy individuals, 4.2% failed to get reproducible FEV_1_ and 9% failed to get reproducible FVC, Finally, one study made in Poland found that 3.5% of subjects fail to achieve repeatability criteria in FVC and 13.6% in FEV_1_ [29]. In our population-based sample, we found that the percentage of failure was 15.7% for FEV_1_ and 22% for FVC.

It is not entirely clear what may be the reason for high rates of poor-quality measurements. One potential reason may be the relatively high number of nurses / technicians conducting the testing; it is well known that technicians play a critical role in obtaining good-quality spirometry [30]. Since the HAPIEE study was conducted in 12 different sites (7 town clinics in the Czech Republic, one clinic in Kaunas, Lithuania, and 4 district clinics in Krakow), there were some 20 field workers involved. We adjusted our results for study area and found that some areas had a significant effect on the odds of poor-quality spirometry. For instance, compared to individuals examined in centers in Poland, individuals examined at Hradec center and had higher odds (OR: 2.26, CI: 1.80–2.81) of providing a poor-quality spirometry test, while individuals examined at Usti nad Labem center had lower odds (OR: 0.70, CI: 0.58–0.84). A strict adherence to study protocol may have been imperfect, despite the fact that all personnel were trained and supervised as much as practically possible. We were unable to include technician ID in the analysis because this information was not available in our data. In addition, a small variation of the spirometry estimates can be explained by the quality of the spirometry device. A study of 34 patients found that 2% of the variation in spirometry measurements was dependent on small flow-sensing devices versus volume-sensing spirometers [31].

Several other limitations should be considered. First, although we adjusted for many covariates, unmeasured confounders may still influence the observed associations. For instance, the number of spirometry tests attempts needed to achieve acceptable and repeatable criteria might be associated with cognitive function, however this information was not available in our dataset. Second, the cognitive assessments used in this study may not fully capture all dimensions of cognition, possibly leading to an underestimation of the relationship between cognitive function and spirometry quality. We suggest that the association between cognitive function and poor-quality spirometry needs more research, mainly including more domains of cognitive function. Finally, the utility of poor-quality spirometry as a biomarker of earlier mortality is uncertain and should be confirmed or refuted in other populations.

Our findings have both clinical and public health implications. Clinically, our findings highlight the need for support and supervision during spirometry testing, especially among older adults. Strategies might include cognitive screening beforehand to identify individuals at risk of inadequate test performance. Additionally, technician training could include strategies to improve test performance in people with impaired cognition and, where possible, to adapt the techniques accordingly. However, the observation that poor-quality spirometry was not linked to increased mortality may reduce concerns about the clinical consequences of poor-quality spirometry in healthy individuals. Still, poor-quality spirometry might be an indicator of broader functional decline. From a public health perspective, population aging is likely to increase the number of individuals with cognitive impairments. Ideally, alternative lung function tests that are less cognitively demanding should be considered in people with cognitive decline.

## Conclusions

In conclusion, our study suggests that lower cognitive function is associated with both worse lung function (impaired spirometry) and poor-quality spirometry. While our results do not provide evidence that excluding poor-quality spirometry may not introduce a major bias from analyses of mortality, the association between cognitive function and the ability to correctly complete spirometry may still affect studies of respiratory functions. These findings underscore the need for robust spirometry testing protocols that would minimize the proportion of low-quality measurements. Further studies should explore poor-quality spirometry testing, as it might be an indicator of impaired cognitive function and may potentially predict earlier mortality.

## Electronic supplementary material

Below is the link to the electronic supplementary material.

Supplementary Material 1

## Data Availability

Data are available upon reasonable request.

## Abbreviations

BMI: Body mass index
CI: Confidence interval
FEV_1_: Forced expiratory volume in the first second
FVC: Forced vital capacity
HAPIEE: Health, Alcohol, and Psychosocial Factors in Eastern Europe study
HR: Hazard ratio
LLN: Lower limit of normality
MMSE: Mini Mental State exam
OR: Odds ratio
