# Supplementary material for: The association between spirometry measurement quality, cognitive function, and mortality
Source: Arch Public Health. 2025 Jul 1;83:170. doi: 10.1186/s13690-025-01660-x (PMC12211210; doi:10.1186/s13690-025-01660-x)
Supplement: Supplementary file 1 — Supplementary Material 1 [file 13690_2025_1660_MOESM1_ESM.docx]

**Additional file 1.** Spearman correlation of cognitive function tests from participants of the HAPIEE study.

|  | Range | Median | IQR | 1 | 2 | 3 | 4 | 5 | 6 |
| --- | --- | --- | --- | --- | --- | --- | --- | --- | --- |
| 1. Immediate verbal memory 1 | 0-10 | 6 | 2 | 1 |  |  |  |  |  |
| 2. Immediate verbal memory 2 | 0-10 | 8 | 3 | 0.65 | 1 |  |  |  |  |
| 3. Immediate verbal memory 3 | 0-10 | 8 | 2 | 0.56 | 0.69 | 1 |  |  |  |
| 4. Delayed verbal memory | 0-10 | 8 | 3 | 0.55 | 0.66 | 0.73 | 1 |  |  |
| 5. Semantic and verbal fluency | 0-66 | 21 | 9 | 0.36 | 0.36 | 0.35 | 0.36 | 1 |  |
| 6. Speed and concentration | 0-90 | 17 | 6 | 0.28 | 0.27 | 0.25 | 0.27 | 0.38 | 1 |
| Cognitive function tests transformed into z-scores, mean=0, standard deviation=1. | | | | | | | | | |

**Additional file 2.** Full results of regression analyses. Multinomial logistic regression for impaired and poor-quality (with respect to healthy spirometry). Logistic regression for poor-quality spirometry (with respect to good-quality spirometry)

|  | **Multinomial logistic regression** | | | | **Logistic regression** | |
| --- | --- | --- | --- | --- | --- | --- |
|  | Impaired vs healthy spirometry | | Poor-quality vs healthy spirometry | | Poor vs good quality spirometry | |
|  | OR (95% CI)^a^ | p-value | OR (95% CI)^b^ | p-value | OR (95% CI)^b^ | p-value |
| **Model 2** | | | | | | |
| Age, years | 1.00 (0.98 – 1.00) | =0.57 | 1.00 (0.99 – 1.00) | =0.91 | 1.00 (0.99 – 1.00) | 0.71 |
| Sex (female) | 0.96 (0.81 – 1.11) | =0.55 | 0.72 (0.66 – 0.79) | <0.001 | 0.72 (0.66 – 0.79) | <0.001 |
| BMI, kg/m^2^ | 1.07 (1.06 – 1.08) | <0.001 | 1.01 (1.00 – 1.02) | <0.001 | 1.01 (0.99 – 1.01) | 0.05 |
| Smoking (yes) | 1.68 (1.43 – 1.95) | <0.001 | 0.91 (0.83 – 1.00) | =0.05 | 0.86 (0.78 – 0.94) | =0.001 |
| Hosp. stroke (yes) | 1.63 (1.20 – 2.22) | =0.001 | 1.36 (1.10 – 1.66) | =0.004 | 1.25 (1.02 – 1.53) | =0.02 |
| Hosp. respiratory disease (yes) | 2.13 (1.81 – 2.50) | <0.001 | 1.05 (0.93 – 1.17) | =0.40 | 0.94 (0.83 – 1.04) | =0.23 |
| Education |  |  |  |  |  |  |
| Primary | Ref. |  | Ref. |  | Ref. |  |
| Secondary and Vocational | 0.67 (0.55 – 0.81) | <0.001 | 0.87 (0.76 – 0.99) | =0.04 | 0.93 (0.81 – 1.05) | =0.27 |
| Higher than secondary | 0.61 (0.48 – 0.76) | <0.001 | 0.90 (0.78 – 1.03) | =0.14 | 0.96 (0.84 – 1.10) | =0.60 |
| Cognitive function |  |  |  |  |  |  |
| First quartile | Ref. |  | Ref. |  | Ref. |  |
| Second quartile | 0.95 (0.78 – 1.15) | =0.63 | 0.96 (0.84 – 1.08) | =0.49 | 0.97 (0.86 – 1.09) | =0.60 |
| Third quartile | 0.74 (0.60 – 0.91) | =0.005 | 0.74 (0.65 – 0.84) | <0.001 | 0.77 (0.68 – 0.87) | <0.001 |
| Fourth quartile | 0.74 (0.58 – 0.92) | =0.009 | 0.76 (0.66 – 0.87) | <0.001 | 0.79 (0.69 – 0.90) | =0.001 |
| Study area |  |  |  |  |  |  |
| Poland | Ref. |  | Ref. |  | Ref. |  |
| Lithuania | 0.36 (0.30 – 0.43) | <0.001 | 0.84 (0.74 – 0.94) | =0.004 | 0.97 (0.85 – 1.08) | =0.56 |
| Hradec | 0.56 (0.35 – 0.89) | =0.01 | 2.05 (1.63 – 2.57) | <0.001 | 2.25 (1.80 – 2.81) | <0.001 |
| Jihlava | 0.72 (0.53 – 0.97) | =0.03 | 0.82 (0.66 – 1.01) | =0.07 | 0.87 (0.70 – 1.07) | =0.20 |
| Karvina | 0.68 (0.51 – 0.91) | =0.01 | 1.00 (0.81 – 1.22) | =0.98 | 1.06 (0.87 – 1.30) | =0.50 |
| Liberec | 0.48 (0.33 – 0.68) | <0.001 | 0.76 (0.61 – 0.94) | =0.01 | 0.85 (0.68 – 1.05) | =0.15 |
| Usti nad Labem | 0.59 (0.45 – 0.76) | <0.001 | 0.65 (0.53 – 0.77) | <0.001 | 0.70 (0.58 – 0.84) | <0.001 |
| Model 2 mutually adjusted for all covariates. OR (95% CI): Odds Ratio at 95% Confidence Interval. ^a^ Odds of impaired lung function. ^b^ Odds of poor-quality spirometry | | | | | | |

**Additional file 3.** Full (n=18,263) vs. complete case analysis (n=12,087) samples; hazard ratios of mortality by spirometry categories.

|  | **Full sample (n=18,263)** | | | | **Complete case analysis (n=12,087)** | | | |
| --- | --- | --- | --- | --- | --- | --- | --- | --- |
|  | No. of deaths | Person years of follow-up | Deaths per 1000 person-years | HR (95% CI)^a^ | No. of deaths | Person years of follow-up | Deaths per 1000 person-years | HR (95% CI)^a^ |
| Healthy spirometry | 1,855 | 147,459 | 12.58  (12.02 – 13.16) | 1.00 | 1,430 | 95,701 | 14.94  (14.18 – 15.73) | 1.00 |
| Impaired spirometry | 627 | 23,212 | 27.01  (24.97 – 29.21) | 1.98  (1.80 – 2.16) | 360 | 11,258 | 31.97  (28.83 – 35.45) | 2.07  (1.84 – 2.33) |
| Poor-quality spirometry | 1151 | 69,776 | 16.49  (15.56 – 17.47) | 1.20  (1.11 – 1.29) | 806 | 42,908 | 18.78  (17.53 – 20.12) | 1.15  (1.05 – 1.25) |
| ^a^ Model adjusted for age and sex. | | | | | | | | |

**Additional file 4. Sensitivity analysis.** Individual domains of cognitive function associated with spirometry categories. Odds ratios of impaired vs. healthy spirometry, poor-quality vs healthy spirometry, and poor- vs good-quality spirometry.

|  | **Immediate verbal memory**  OR (95% CI) | | | | **Delayed verbal memory**  OR (95% CI) | | | | **Semantic and verbal fluency**  OR (95% CI) | | | | **Speed and concentration**  OR (95% CI) | | | |
| --- | --- | --- | --- | --- | --- | --- | --- | --- | --- | --- | --- | --- | --- | --- | --- | --- |
|  | First quartile | Second quartile | Third quartile | Fourth quartile | First quartile | Second quartile | Third quartile | Fourth quartile | First quartile | Second quartile | Third quartile | Fourth quartile | First quartile | Second quartile | Third quartile | Fourth quartile |
| **Multinomial regression** (healthy spirometry is the reference group) | | | | | | | | | | | | | | | | |
| Impaired vs. healthy spirometry | Ref. | 0.78 (0.65 – 0.93) | 0.61 (0.50 – 0.74) | 0.62 (0.50 – 0.77) | Ref. | 0.84 (0.71 – 0.99) | 0.73 (0.58 – 0.90) | 0.71 (0.56 – 0.91) | Ref. | 1.05 (0.87 – 1.26) | 0.89 (0.74 – 1.07) | 0.85 (0.68 – 1.06) | Ref. | 1.04 (0.87 – 1.25) | 0.86 (0.70 – 1.05) | 0.89 (0.72 – 1.09) |
| Poor-quality vs. healthy spirometry | Ref. | 0.99 (0.88 – 1.11) | 0.84 (0.74 – 0.95) | 0.81 (0.71 – 0.92) | Ref. | 0.85 (0.77 – 0.92) | 0.81 (0.71 – 0.92) | 0.85 (0.74 – 0.98) | Ref. | 0.78 (0.70 0.88) | 0.82 (0.73 – 0.92) | 0.86 (0.77 0.98) | Ref. | 0.91 (0.81 – 1.02) | 0.91 (0.81 – 1.02) | 0.87 (0.77 – 0.98) |
| **Logistic regression** (good-quality spirometry is the reference group) | | | | | | | | | | | | | | | | |
| Poor- vs. good-quality spirometry | Ref. | 1.03 (0.92 – 1.15) | 0.89 (0.79 – 1.00) | 0.86 (0.75 – 0.97) | Ref. | 0.87 (0.79 – 0.96) | 0.84 (0.74 – 0.95) | 0.89 (0.77 – 1.01) | Ref. | 0.78 (0.70 – 0.87) | 0.83 (0.74 – 0.93) | 0.88 (0.78 – 0.99) | Ref. | 0.91 (0.81 – 1.01) | 0.93 (0.83 – 1.04) | 0.88 (0.79 – 0.99) |
| All models were adjusted for age, sex, study area, BMI, smoking status, education, hospitalized/diagnosed for respiratory disease, and hospitalized/diagnosed for stroke. | | | | | | | | | | | | | | | | |
